# Glioblastoma Phagocytic Cell Death: Balancing the Opportunities for Therapeutic Manipulation

**DOI:** 10.3390/cells13100823

**Published:** 2024-05-11

**Authors:** Ruochen Du, Shashwat Tripathi, Hinda Najem, Daniel J. Brat, Rimas V. Lukas, Peng Zhang, Amy B. Heimberger

**Affiliations:** 1 Department of Neurological Surgery, Feinberg School of Medicine, Northwestern University, Chicago, IL 60611, USA; ruochen.du@northwestern.edu (R.D.); shashwat.tripathi@northwestern.edu (S.T.); hinda.najem@northwestern.edu (H.N.); peng@northwestern.edu (P.Z.); 2 Malnati Brain Tumor Institute of the Robert H. Lurie Comprehensive Cancer Center, Feinberg School of Medicine, Northwestern University, Chicago, IL 60611, USA; rimas.lukas@nm.org; 3 Department of Pathology, Feinberg School of Medicine, Northwestern University, Chicago, IL 60611, USA; daniel.brat@northwestern.edu; 4 Department of Neurology, Feinberg School of Medicine, Northwestern University, Chicago, IL 60611, USA

**Keywords:** gliomas, myeloid cells, immune checkpoint blockade

## Abstract

Macrophages and microglia are professional phagocytes that sense and migrate toward “eat-me” signals. The role of phagocytic cells is to maintain homeostasis by engulfing senescent or apoptotic cells, debris, and abnormally aggregated macromolecules. Usually, dying cells send out “find-me” signals, facilitating the recruitment of phagocytes. Healthy cells can also promote or inhibit the phagocytosis phenomenon of macrophages and microglia by tuning the balance between “eat-me” and “don’t-eat-me” signals at different stages in their lifespan, while the “don’t-eat-me” signals are often hijacked by tumor cells as a mechanism of immune evasion. Using a combination of bioinformatic analysis and spatial profiling, we delineate the balance of the “don’t-eat-me” CD47/SIRPα and “eat-me” CALR/STC1 ligand–receptor interactions to guide therapeutic strategies that are being developed for glioblastoma sequestered in the central nervous system (CNS).

## 1. Introduction

Gliomas are primary intracranial neoplasms that arise from specific glial cells including astrocytes, oligodendrocytes, and ependymal cells [1]. High-grade gliomas are characterized by rapid progression, frequent recurrence, and poor prognosis. They typically present with symptoms of headaches, focal neurological deficits, or cognitive impairment. The diagnostic criteria for gliomas have undergone an evolution from standard histological characterization to one that heavily incorporates molecular and genetic classification. The presence or absence of mutation of genes encoding isocitrate dehydrogenase 1 or 2 (IDH) defines the types of adult-infiltrating gliomas. Broadly, the diffuse gliomas of adults are categorized as IDH-mutant astrocytoma, oligodendroglioma (also harboring IDH mutations), or IDH wildtype (IDHwt) glioblastoma [2].

Surgery, radiation, chemotherapy, and alternating electrical fields are therapeutic options for diffuse gliomas [3]. However, these modalities, alone or in combination, are not curative and here is a substantial unmet need for therapeutic advances. Spurred on by the success observed in other malignancies, immunotherapeutic investigations have been actively pursued [4,5]. Unfortunately, thus far, they have not impacted the survival of most glioma patients [6]. There are likely many reasons for this. Gliomas are immunosuppressive and typically lack effector immune responses. The most frequent glioma-infiltrating immune cells are myeloid cells, which include myeloid-derived suppressor cells, monocytes, microglia, bone marrow-derived macrophages (BMDM), and dendritic cells (DCs)—a heterogeneous population in both phenotype and function [7]. All of these can be “tuned” towards favorable or unfavorable behavior within the tumor. Macrophages and microglia can be polarized to either pro-inflammatory (M1-like) or immune-suppressive (M2-like) states and a lower M1/M2 ratio correlates with a worse prognosis in gliomas [8,9]. Our understanding is further complicated by the dynamic nature of these functional changes. The molecular characteristics of myeloid cells change in the evolving tumor microenvironment (TME) associated with disease progression. To either block or reverse impairments in phagocytic functions, a variety of approaches focused on targeting phagocytosis could be considered.

## 2. Blockade of the “Don’t-Eat-Me” CD47/SIRPα Axis

CD47, a glycosylated type I transmembrane protein belonging to the immunoglobulin superfamily, is expressed on normal cells in the body and serves as a “don’t-eat-me” signal [10,11]. CD47 prevents detection and elimination by immune cells by engaging with the inhibitory receptor signal regulatory protein alpha (SIRPα), which is expressed on myeloid cells such as macrophages, DCs, and their precursors [11,12]. When molecular signals that inhibit phagocytosis, such as CD47, are degraded, there is an increase in macrophage-mediated phagocytosis of dying cells [13]. The SIRPα cytoplasmic domain contains two immunoreceptor tyrosine inhibitory motifs (ITIM). The binding of CD47 with SIRPα on macrophages triggers ITIM phosphorylation and the downstream recruitment of Src homology region 2 (SH-2) domain-containing phosphatase (SHP)-1/2, thereby suppressing both phagocytic function and inflammatory signaling [14,15,16]. 

**Figure 1:**
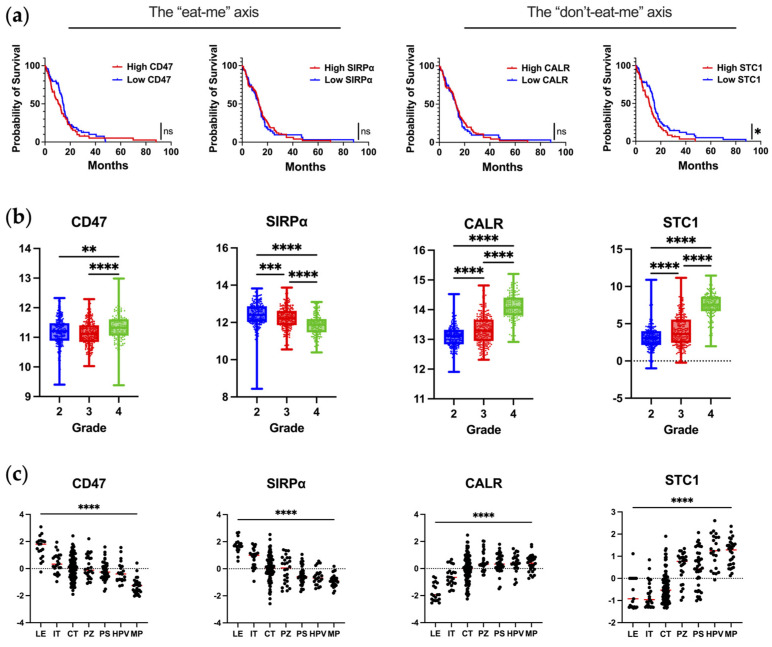
The expression of key molecules regulating phagocytosis in human glioblastoma. (**a**) Correlation of mRNA expression with overall survival in newly diagnosed IDHwt glioblastoma patients as analyzed from TCGA database using Gliovis. Kaplan–Meier curve is calculated based on high versus low expression of markers dichotomized based on the median expression in glioma patients. CD47: *n* = 142; median survival for high–CD47 (*n* = 70) and low–CD47 (*n* = 72) are 11.0 and 14.5 months, respectively (*p* = 0.24). SIRPα: *n* = 145; median survival for high–SIRPα (*n* = 74) and low–SIRPα (*n* = 71) are 13.3 and 13.0 months, respectively (*p* = 0.82). CALR: *n* = 142; median survival for high–CALR (*n* = 71) and low–CALR (*n* = 71) are 13.1 and 13.0 months, respectively (*p* = 0.88). STC1: *n* = 142; median survival for high–STC1 (*n* = 70) and low–STC1 (*n* = 72) are 10.8 and 14.9 months, respectively (*p* = 0.03). Mantel–Cox test was used to compare groups: ns denotes *p* > 0.05, * denotes *p* ≤ 0.05. (**b**) Expression of markers at the mRNA level as a function of glioma grade as analyzed from TCGA database using Gliovis. Ordinary one–way ANOVA with Tukey’s multiple comparison test was used to compare each grade: * denotes *p* ≤ 0.05, ** denotes *p* ≤ 0.01, *** denotes *p* ≤ 0.001, **** denotes *p* ≤ 0.0001. (**c**) Expression of markers based on anatomical location as analyzed from the Ivy Atlas using Gliovis. Ordinary one–way ANOVA with Tukey’s multiple comparison test was used to compare locations: **** denotes *p* ≤ 0.0001. LE, leading edge; IT, infiltrating tumor; CT, cellular tumor; PZ, perinecrotic zone; PS, pseudopalisading cells around necrosis; HPV, hyperplastic blood vessels in cellular tumor; MP, microvascular proliferation. The markers are annotated with the axes they are part of, respectively.

Almost all types of tumor cells upregulate CD47 on malignant cells [12,17]. In glioblastoma, CD47 overexpression has been reported to be associated with poor progression-free and overall survival [12]. However, this observation was not validated in our query of publicly available datasets of scRNA-seq of newly diagnosed IDHwt glioblastoma (Figure 1a). In the analysis of CD47/SIRPα as a function of WHO grade, SIRPα expression is significantly reduced in grade 4 gliomas (Figure 1b). The downregulation of SIPRα by macrophages as they infiltrate into the glioblastoma TME could represent a novel mechanism of immune evasion that has not yet been described. To evaluate this hypothesis, matched peripheral blood and glioblastoma-infiltrating macrophages could be profiled to determine if there is a difference in the mean fluorescent intensity of SIRPα expression.

**Figure 2:**
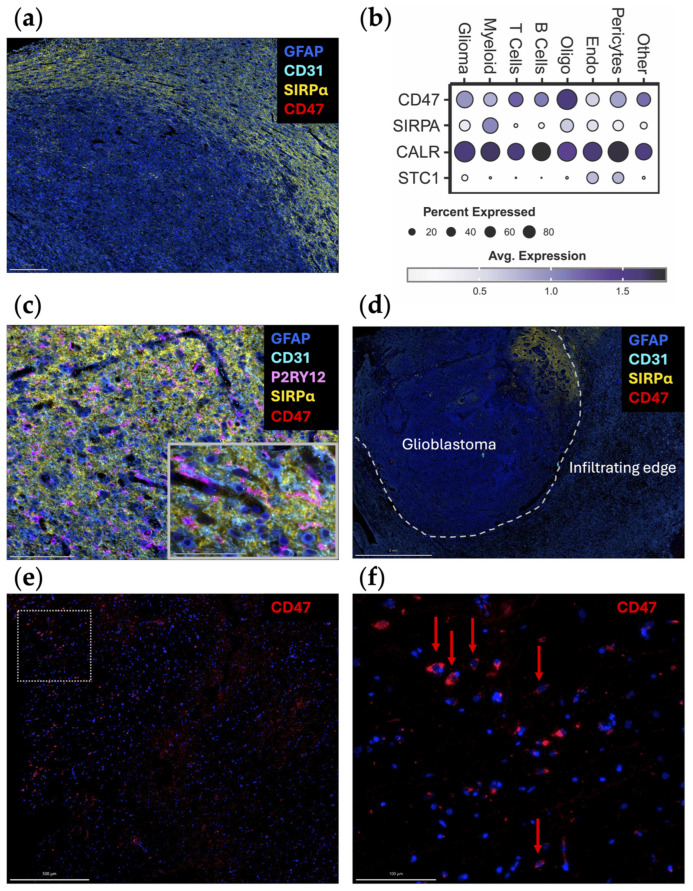
Original representative images of the spatial and cellular localization of CD47/SIRPα expression. (**a**) A newly diagnosed glioblastoma specimen resected as a lobectomy in continuity with the adjacent infiltrating brain was analyzed using automated sequential multiplex immunofluorescence (*n* = 2) as previously described [21,22,23]. SIRPα (yellow; Cell Signaling, clone D613M, dilution 1/100) is expressed at the leading edge. CD47 (red; Novus Bio, clone B6H12.2, dilution 1/100) is expressed at minimal levels. Scale bar = 200 µm. (**b**) Dot plot displaying marker expression within gliomas analyzed with the scRNA seq dataset from Abdelfattah et al. [24]. Bubble size corresponds to the percentage of cells expressing gene marker; colors indicate average expression. Glioma, glioma tumor cells and astrocytes; oligo, oligodendrocytes; endo, endothelial cells. (**c**) Adjacent infiltrating region of the brain analyzed using automated sequential multiplex immunofluorescence as previously described with magnified insert [21,22,23]. SIRPα (yellow) is seen in association with microglia (P2RY12; Atlas Antibodies, polyclonal, dilution 1/1000). Scale bar = 100 µm (main panel) and 50 µm (insert). (**d**) Newly diagnosed glioblastoma sample with adjacent peritumoral brain (lobectomy resection) analyzed using automated sequential multiplex immunofluorescence as previously described [21,22,23]. SIRPα (yellow) expression is confined to a focal area at the edge. Dotted line represents the edge between the tumor and the adjacent brain. Scale bar = 2 mm. (**e**) Positive control multiplex immunofluorescence images of a glioblastoma expressing CD47 (red) within the tumor region with panel (**f**) representing the magnified window in (**e**) as highlighted with the dotted box. Red arrows denote rare cells expressing CD47. The other markers were turned off to see the CD47 expression more clearly. Scale bars = 500 µm (**e**) and 100 µm (**f**). Nuclei are denoted with DAPI (dark blue, ThermoFisher Scientific); glioblastoma tumor cells and astrocytes are marked with GFAP (light blue; Sigma, clone GA5, dilution 1/2000); vasculature is marked with CD31 (cyan blue; Abcam, clone EPR17259, dilution 1/1500); microglia are marked with P2RY12 (pink).

When the spatial distribution of CD47 and SIRPα mRNA is assessed within glioblastoma, both CD47 and SIRPα are expressed to a greater extent at the leading edge (Figure 1c), indicating this may be a mechanism for immune evasion as tumor cells infiltrate the brain. A future direction of investigation should include ascertaining if there are higher levels of CD47 expression in widely disseminated cases of diffusely infiltrating gliomas (i.e., similar to gliomatosis cerebri) relative to more conventional cases of glioblastoma. An original representative image of the multiplex imaging of glioblastoma resected as a lobectomy in continuity with the adjacent infiltrating brain shows that SIRPα expression is particularly prominent at the leading-edge/infiltrating region (Figure 2a). Bioinformatic analyses of scRNA seq datasets of eighteen glioma patients (two with low-grade glioma, eleven with newly diagnosed glioblastoma, and five with recurrent glioblastoma) demonstrate that SIRPA is expressed in myeloid cells (Figure 2b), and multiplex imaging shows P2RY12^+^ microglia also express SIRPα (Figure 2c) [18,19]. Notably, SIRPα is heterogeneously expressed at the invasive edge (Figure 2d). Given this marked heterogeneity, SIRPα monotherapies are unlikely to exert marked therapeutic benefits, especially in scenarios in which super total resections that include the invasive edge are removed from the patient. The expression of CD47 is rare (Figure 2e,f), suggesting that the CD47 and SIRPα protein interaction is not typical in newly diagnosed glioblastoma. However, because CD47 expression is increased at recurrence [20], this interaction should be further investigated in this clinical and pathological setting.

Anti-CD47 monoclonal antibodies (mAbs) promote the antigen presentation function of macrophages and subsequently prime cytotoxic CD8^+^ T cells in co-culture experiments [25]. Several studies have indicated anti-CD47 therapeutics may be a viable strategy for glioma. In vitro analysis of mouse BMDM and peripheral blood-derived human macrophages have shown that the addition of anti-CD47 mAb enhances myeloid cell effector functions against glioblastoma cells regardless of the M1/M2 polarization status [26]. Additionally, anti-CD47 mAbs enabled human peripheral blood-derived macrophages to phagocytose patient-derived glioblastoma neurospheres in vitro, which was corroborated by decreased tumor growth in an intracranial xenograft model of glioblastoma [12]. Using murine models with genetically color-coded macrophages (*Ccr2*^RFP^) and microglia (*Cx3cr1*^GFP^), both populations were found to participate in anti-CD47 enhanced tumor cell phagocytosis in vivo [18]. In a human glioblastoma xenograft model, anti-CD47 induced M1 polarization of macrophages within the TME and prolonged survival [26]. mIAP301, an anti-CD47 mAb that blocks the binding between murine CD47 and SIRPα, has been shown to enhance phagocytosis of GL261 glioma cells and glioma stem cells by macrophages and prolonged mouse survival [27,28]. In pediatric high-grade brain tumors, Magrolimab (Hu5F9-G4), a humanized anti-CD47 antibody, exhibited therapeutic effectiveness in patient-derived orthotopic xenograft models when administered intraperitoneally with minimal impact on normal human neural cells [29]. A key consideration is how closely these models recapitulate the biology of human glioblastoma from both the perspective of myeloid infiltration and location and that of CD47 expression levels and heterogeneity. A companion biomarker will likely be needed to enrich patients who would benefit from a CD47/SIRPα strategy. Furthermore, the large molecular size of anti-CD47 mAbs will likely hinder penetration into the glioblastoma TME. To overcome this issue, smaller nanobodies could be considered [30]. However, this strategy has not yet been tested in glioma preclinical models. Alternatively, approaches to overcome the blood–brain barrier (BBB) could be considered [31].

Although anti-CD47 showed promising results in preclinical studies, their therapeutic effects could be limited by the pattern of CD47 expression in glioma patients, especially newly diagnosed subjects. Contrary to clonotypic homogeneous glioma cell lines where CD47 is consistently highly expressed, newly diagnosed glioblastoma specimens exhibited heterogeneous and very low expression of CD47 (Figure 2) [32]. Assessing CD47 expression of 75 human glioma specimens of different grade with immunohistochemical staining revealed that less than 30% tumor cells are CD47-positive in grade 4 gliomas, and the percentage is even lower in low-grade gliomas [32]. As such, the experimental models likely overestimate the therapeutic effect. Further confounding this issue is that even within these clonotypic models, there is significant heterogeneity in anti-CD47 antibody binding [20]. One potential strategy for overcoming this limitation is to use anti-CD47 treatment as a combination with standard-of-care therapy. This proof of principal strategy has been shown to be viable for STING agonist-loaded, CD47/PD-L1 targeting nanoparticles [33].

Because CD47 is ubiquitously expressed throughout the body, the lack of tumor selectivity leads to undesirable side effects such as anemia and thrombocytopenia [34]. Attempts to overcome this limitation have included the development of bispecific antibodies, such as TG-1801 and IMM0306, that target both CD47 and tumor cells, thereby aiming to restrict activity against non-tumor cells [35,36,37]. Nevertheless, these therapeutic approaches suffer from a lower affinity for CD47, thus diminishing drug efficacy. An alternative method for blocking CD47 is the use of recombinant proteins that incorporate a SIRPα extracellular motif fused to the Fc region of the immunoglobulin heavy chain. In addition to higher affinity, the SIRPα fusion protein may offer better penetration into solid tumors relative to anti-CD47 mAb due to their smaller molecular weight. Evorpacept (ALX-148), a CD47-blocking fusion protein, is currently being tested with the PD-1 antibody pembrolizumab in a variety of solid cancers [38]. TTI-622, consisting of the CD47-binding domain of human SIRPα linked to the Fc region of human IgG4, is also in early-phase clinical trials against a variety of malignancies [39,40]. In contrast, antibodies targeting SIRPα (anti-SIRPα) are still under development. ADU-1805 is an early example that demonstrated in vitro efficacy comparable to that of anti-CD47 in promoting macrophage phagocytosis [41]. As the expression of SIRPα is more restricted to certain cell types, anti-SIRPα may be more selective, thereby avoiding some of the side effects associated with anti-CD47 mAbs [41].

Small molecules have been developed to disrupt the CD47-SIRPα axis by interfering with their binding or expression. Glutaminyl-peptide cyclotransferase-like protein (QPCTL) regulates the CD47-SIRPα interaction by facilitating the formation of pyroglutamate (pGlu) on CD47 at the SIRPα binding site [42]. Preclinical studies have identified candidate small-molecule inhibitors of QPCTL, such as luteolin and ISM004-1057D, that suppress the pGlu modification of CD47 and reduce its binding affinity to SIRPα [43,44]. Furthermore, the transcription factor Myc has been identified as a direct regulator of CD47 expression [45]. As a commonly mutated oncogene, several Myc inhibitors have been developed [46,47,48]. One such molecule, RRx-001, has entered clinical trials for newly diagnosed glioblastoma, small cell lung cancer, and other advanced metastatic cancers in combination regimens [46,49,50,51]. As some of these therapies are being explored in trials for CNS malignancies, an iterative process utilizing knowledge gleaned from assessments in preclinical glioma models that more closely approximate human biology would be beneficial. 

## 3. Leveraging the “Eat-Me” CALR/STC1 Axis

**Figure 3:**
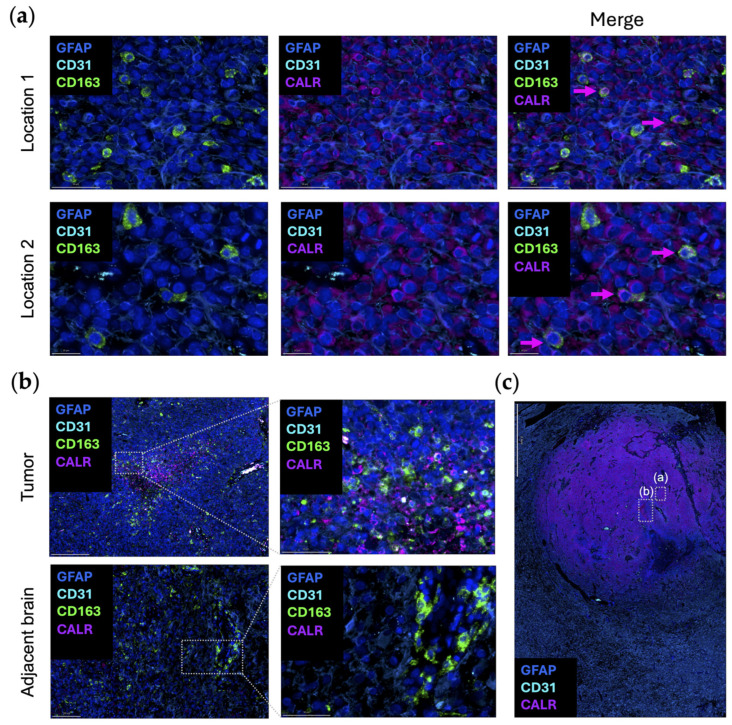
Original representative images of the spatial localization of CALR/STC1 expression. (**a**) Representative images showing the colocalization of CALR (purple; Abcam, clone FMC75, dilution 1/1000) and CD163 (green; Abcam, clone EPR19518, dilution 1/600) within the same cell. Multiple regions were analyzed and show concordant expression of both CD163 (green) and CALR (purple). Location 2 is highlighted in panel (**c**) with boxed window “(a)”. Scale bar = 50 µm (location 1) and 20 µm (location 2). (**b**) CALR (purple) colocalized with CD163 (green) in the perinecrotic zone within the glioblastoma (top panels). This area is highlighted in panel (**c**) within boxed window “(b)”. In the region of the adjacent brain, CD163 (green) expression is present in the blood vessels, but CALR (purple) is absent (bottom panels). Panels on the right are magnified views of the highlighted area on the left. Scale bar = 200 µm (top left), 50 µm (top right), 100 µm (bottom left), and 50 µm (bottom right). (**c**) Newly diagnosed glioblastoma sample with adjacent peritumoral brain (lobectomy resections, *n* = 2) analyzed using automated sequential multiplex immunofluorescence as previously described [21,22,23]. CALR (purple) is expressed throughout the tumor region. Area shown in boxed window “(a)” is location 2 of panel (**a**). Area shown in boxed window “(b)” is tumor in panel (**b**). Scale bar = 2 mm. Nuclei are denoted with DAPI (dark blue; ThermoFisher Scientific); glioblastoma tumor cells and astrocytes are marked with GFAP (light blue; Sigma, clone GA5, dilution 1/2000); vasculature is marked with CD31 (cyan blue; Abcam, clone EPR17259, dilution 1/1500); macrophages are marked with CD163 (green).

Dying cells can promote macrophage phagocytosis by exposing damage-associated molecular patterns. Calreticulin (CALR) typically resides in the endoplasmic reticulum (ER), where it acts as a luminal Ca(2+)-buffering chaperone [52]. CALR is also involved in the folding of newly synthesized proteins, preventing the exportation of misfolded proteins from the ER [52]. In terms of the immune response, CALR directly affects antigen presentation to cytotoxic T cells by participating in the folding of a major histocompatibility complex class I molecule [53]. As one of the canonical “eat-me” signaling molecules, when CALR is expressed on the outer leaflet of the cellular membranes of dying cells and living cancer cells, phagocytosis is initiated by interacting with complement protein C1q or low-density lipoprotein receptor-related protein 1 (LRP1) on macrophages [54,55]. CD47 blocks CALR-mediated phagocytosis and is upregulated when there are high levels of CALR expression on cancer cells [55,56]. When CALR is over-expressed in glioma cells, there is an increased propensity for radiation-induced apoptosis through the suppression of AKT signaling [57]. To date, there has not been an analysis of CALR expression in newly diagnosed gliomas relative to post radiation to ascertain if CALR is increased. It is uncommon to obtain tissue samples from humans with CNS tumors immediately post radiation as there is typically no clinical indication for surgery at that timepoint. In turn, our understanding relies on preclinical models which may not perfectly recapitulate what occurs in humans. Even when cancer cells do not display CALR, studies have shown that macrophages themselves can secrete and/or display CALR to guide their phagocytosis [58]. This observation was validated in an original representative image of the multiplex imaging of glioblastoma (Figure 3a). The expression of CALR by CD163^+^ macrophages is seen in the tumor area, but not in the adjacent brain (Figure 3b). 

Besides macrophages, CALR signaling is utilized to promote natural killer (NK) cell function via NKp46, an ancient activating receptor expressed in NK cells that recognizes externalized CALR [59,60]. One could consider trying to increase the delivery of NK cells to the TME to further engage with CALR, which has been evaluated in preclinical models of infection [59]. A strategy for modeling this type of preclinical assessment would be the co-culture of ex vivo resected tumors with autologous NK cells to ascertain if the mere presence of an increasing NK cell population would be sufficient for clearance of CALR-expressing tumor cells. Upregulation of CALR could also be modeled in this type of assay with the addition of either ruxolitinib or azacitidine. This could then be followed up with in vivo preclinical models in immune-competent background with NK delivery enhanced with BBB opening. However, in the indication of glioblastoma, we have previously shown that NK cells become irreversibly dysfunctional, and this strategy would require targeting TGF-β [61]. In other types of CNS gliomas, immune suppression may not be so limiting.

Stanniocalcin-1 (STC1) is a glycoprotein that was first identified as a regulator of calcium homeostasis and that promotes tumor cell stemness, metastasis, proliferation, and chemoresistance [62,63,64,65]. STC1 has more recently been described as an immune checkpoint that prevents macrophages from consuming dying cells. The interaction of CALR and STC1 blocks CALR exposure, and consequently macrophages are unable to consume the dying cell and perform antigen presentation [66]. STC1 is a prognostic marker in gliomas (*p* = 0.03), agreeing with a previous analysis (Figure 1a) [67]. Mechanistically, STC1 maintains glioblastoma “stemness” through the NOTCH1-SOX pathway and enhances tumorigenesis in vivo [65]. The migration and invasion of glioblastoma cells are partially regulated by STC1 through the TGF-β/SMAD4 signaling pathways [68]. Proof-of-concept genetic knock-down of STC1 decreases cancer cell growth, and several miRNAs targeting STC1, such as miR-606 and miR-146-5p, have been exploited for therapeutic purposes in preclinical models [69,70,71].

Using publicly available datasets of scRNA seq of glioblastoma, both CALR and STC1 expression are increased as a function of glioma grade (Figure 1b). This is in line with the theme that the higher the grade of tumor or the later the disease state, the more immunosuppressive the TME becomes. In contrast to the localization of CD47/SIRPα at the leading edge, CALR/STC1 is present throughout the TME (Figure 1c). For CALR, expression is especially high near hyperplastic vessels and microvascular proliferation. These mRNA data would imply that the CALR/STC1 interaction is perivascular. Spatial multiplex imaging of glioblastoma resected as a lobectomy in continuity with the adjacent infiltrating brain shows that CALR is markedly expressed throughout the TME (Figure 3c). This perivascular process could be part of what impedes the activity of peripheral immune cells trafficking to the tumor in the CNS.

Relative to the CD47/SIRPα axis, there are fewer strategies for modulating the CALR/STC1 axis. Some strategies aim to promote the “eat-me” signal while blocking the “don’t-eat-me” signal. In blood malignancies, ruxolitinib and azacitidine have been found to induce endogenous overexpression of the pro-phagocytic cell membrane CALR [72,73]. When tested in combination with the anti-CD47 monoclonal antibody magrolimab, the combination enhanced anti-tumor effects compared to monotherapy. However, this improvement was only moderate, likely due to the redundancy of their downstream effects on macrophages. Another strategy might be in combination with pro-apoptotic agents. The triple combination of azacitidine, venetoclax, and magrolimab showed a tolerable safety profile with an encouraging complete remission rate in acute myeloid leukemia [74]. However, compared to blood cancers, gliomas pose more challenges to these combination strategies due to their unique physical environment and intratumoral heterogeneity, which must be leveraged with novel drug design, drug delivery methods, and drug schedules. Azacitidine has been used previously both in preclinical models and in human subjects with glioblastoma and may be a starting point for combinatorial strategies given the prior experience. JNJ-88549968 is a T cell redirecting bispecific antibody that selectively targets CALR mutations (CALRmut) that is being evaluated in myeloproliferative neoplasms (NCT06150157) [75]. However, based on TCGA, there are no CALRmut in glioblastoma. At this time, there are no clinical trials targeting STC1.

## 4. Discussion

The CD47-SIRPα axis represents a novel target for treating glioblastoma based on the encouraging outcomes in preclinical models of gliomas. However, a key consideration is the heterogeneity and low expression of this target in many solid cancers. Anti-CD47 therapy has demonstrated promise preclinically in glioma models and is the only immunotherapy in clinical trials, but its effectiveness will likely be limited due to variable CD47 expression in glioblastoma patients, especially in newly diagnosed cases. For tumor-expressed CD47, tumor delivery strategies would need to be considered given the low BBB penetrance of antibodies. CD47 is commonly found on healthy cells throughout the body, and therefore there is a theoretical risk of inducing off-target toxicities when treating with an anti-CD47 strategy. Approaches which allow for trapping of the therapeutic target in the CNS may have value [76,77]. SIRPα is diffusely expressed throughout the brain, so it is uncertain what this off-target binding would induce. To increase the therapeutic index, bispecific antibodies or antibody–drug conjugates could be considered to improve the target specificity of CD47 and SIRPα neutralization agents. It will also be necessary to identify a common yet selective cell surface marker for glioma cells and/or glioma-associated macrophages/microglia. Timing over the disease course, something which is difficult to model in animals with short survival, may also prove to be an important consideration. In the case of anti-CD47 strategies, given the higher expression at recurrence, clinical trials would need to focus on the recurrent patient with a companion biomarker to identify the appropriate patient population.

Relative to CD47, the expression of CALR may be more prevalent in glioblastoma. However, strategies that require upregulation are more pharmaceutically challenging relative to blocking the function of a ligand/receptor pair with an inhibitory or antagonistic drug. Even with signals of response in blood cancers, neither ruxolitinib nor azacitidine were originally designed to amplify the expression of “eat-me” signals. In this case, non-coding RNAs that regulate gene expression and mRNA stability might be a viable approach to explore. However, RNA-based regulatory networks lack cell type specificity to some extent. 

Currently, there are no therapies targeting STC1. Due to the fundamental role of calcium homeostasis in human health, targeting STC1 has safety concerns. Similarly, C1q is the initiator of the classical complement activation pathway, which plays a central role in basic human immunology. LRP1, the other receptor of CALR on phagocytes, has not been studied in gliomas. In Alzheimer’s disease, early evidence has shown that LRP1 activation can facilitate the clearance of amyloid-β, which can be induced with formononetin treatment [78]. As such, LRP1 might represent a promising target in the CNS, but will require more preclinical studies to decipher its role in glioma cancer cell clearance. Because LRP1 is a ubiquitous endocytosis cell surface receptor expressed in several important organs including lung, heart, and kidney and has an important role in tissue healing, targeting LRP1 will require specificity to avoid severe side effects [79].

The spatial distribution of these complementary mechanisms indicates that different administration strategies need to be considered. For example, since the CD47/SIRPα axis resides at the infiltrating margin of glioblastoma, including the adjacent microglia population, these regions typically have an intact BBB. As such, therapeutics targeting these axes may need to consider agents that have BBB capabilities or use strategies that can open the BBB, such as low-intensity pulsed ultrasound [80,81,82]. Alternatively, the therapeutic could be deposited into the surgical cavity post resection but would likely be limited by diffusion. Given the vascular and tumor location of CALR/STC1, systemic administration may suffice but may not reach the infiltrating edge. NK cells expressing NKp46 can also interact with CALR, but NK cells are relatively rare in glioblastoma and typically are confined to the perivascular space—the zone of CXCL9/10/11/12 chemokine support, which would require a strategy to drive these cells deep into the TME alongside a strategy to prevent them from becoming immune suppressed. Cumulatively, more due diligence will be necessary prior to exploiting therapeutics that leverage the balance of innate immune phagocytosis.

## 5. Conclusions

In this review, we examined the role of phagocytic cell death in gliomas and strategies to modulate the balance between “don’t-eat-me” CD47/SIRPα and “eat-me” CALR/STC1 axes for therapeutic benefits. Preclinical studies have shown promising results. However, the expression of these targets is limited and highly heterogeneous within the glioma TME. Moreover, no agent has currently advanced to clinical trials for CNS malignancies. While targeting phagocytic cell death represents a promising avenue for cancer therapy, further research efforts are needed before these approaches can be clinically implemented for the management of gliomas.

## Data Availability

Not applicable.
